# Monoglucosyl-rutin as a potential radioprotector in mammalian cells

**DOI:** 10.3892/mmr.2014.2181

**Published:** 2014-04-24

**Authors:** SHIGEAKI SUNADA, HIROSHI FUJISAWA, IAN M. CARTWRIGHT, JUNKO MAEDA, COLLEEN A. BRENTS, KAZUE MIZUNO, YASUSHI AIZAWA, TAKAMITSU A. KATO, MITSURU UESAKA

**Affiliations:** 1 Department of Nuclear Engineering and Management, The University of Tokyo, Tokyo 113-8656, Japan; 2 Department of Bioengineering, School of Engineering, The University of Tokyo, Tokyo 113-8656, Japan; 3 Department of Environmental and Radiological Health Sciences, Colorado State University, Fort Collins, CO 80523, USA; 4 Center for Disease Biology and Integrative Medicine, Faculty of Medicine, The University of Tokyo, Tokyo 113-8656, Japan; 5 Research and Development Group, Toyo Sugar Refining Co., Ltd., Yoto Bldg., Tokyo 103-0046, Japan; 6 Nuclear Professional School, School of Engineering, The University of Tokyo, Naka, Ibaraki 319-1188, Japan

**Keywords:** radioprotector, monoglucosyl-rutin, sister chromatid exchange, γH2AX

## Abstract

In the present study, the role of monoglucosyl-rutin as a potential radioprotector was investigated using mammalian cell culture models. Cell survival and DNA damage were assessed using colony formation, sister chromatid exchange and γH2AX assays. It was demonstrated that monoglucosyl-rutin was able to increase cell survival when exposed to ionizing radiation, possibly by decreasing the amount of base damage experienced by the cell. However, the present study also demonstrated that, despite monoglucosyl-rutin exhibiting radioprotective effects at low doses, high doses of monoglucosyl-rutin led to a decrease in plating efficiency and an increased doubling time. This effect may be due to double-strand breaks caused by high concentrations of monoglucosyl-rutin.

## Introduction

Ionizing radiation is an important clinical tool for the treatment of cancer. However, it presents potential harmful effects for human health, including acute radiation syndrome, mutagenesis and carcinogenesis. To increase the effectiveness of ionizing radiation it is critical to find a way to mitigate the damaging effect of ionizing radiation on healthy tissues surrounding the tumor. In addition to use with radiotherapy, potential radioprotectors may also be useful for the protection of radiation workers who are exposed to low or moderate levels of radiation on a semi-frequent basis.

When tissues are irradiated with low linear energy transfer (LET) radiation, for example X-rays or γ-rays, DNA is primarily damaged indirectly through free radicals. The free radicals are formed when the ionizing radiation interacts with water within the cells. The free radicals that are formed are highly reactive and easily interact with and damage DNA if they form in a close proximity to the DNA itself. Indirect DNA damage contributes to approximately two thirds of ionizing events during low LET ionizing radiation (1). There are two types of free radicals, short- and long-lived radicals. Long-lived radicals are able to induce DNA lesions, for example DNA base damage, however, do not cause serious lesions, including DNA double-strand breaks. The short-lived radicals are able to induce double-strand breaks and thus are the most damaging and potentially harmful type of free radical (2). Since DNA damage in low LET radiation mainly occurs indirectly, the majority of classical radiation protectors, including dimethyl sulfoxide and WR-2721, are radical scavengers that inhibit reactive oxygen species interacting with DNA (3,4).

Natural flavonoids, such as quercetin and rutin, are known antioxidants (5). Previous studies have suggested that these flavonoids may protect cells and mice from ultra-violet radiation and ionizing radiation (6,7). These flavonoids are extremely insoluble in water, which has prevented in depth studies into their potential use as radioprotectors. In an effort to increase their water solubility, modified flavonoids have been available, and one such flavonoid is monoglucosyl-rutin (αG-rutin PS™) (8,9).

In the present study, the protective effect of monoglucosyl-rutin against radiation was investigated in mammalian Chinese hamster ovary (CHO) 10B2 cells. It was hypothesized that irradiated cells treated with monoglucosyl-rutin would have an increased survival rate when compared with the control cells, and that monoglucosyl-rutin may be a potential free radical scavenger.

## Materials and methods

### Cell culture

CHO10B2 cells were grown in α-minimum essential medium (α-MEM; Invitrogen Life Technologies, Carlsbad, CA, USA) supplemented with 10% fetal bovine serum (FBS; HyClone Laboratories, South Logan, UT, USA), antibiotic-antimycotic (anti-anti; Invitrogen Life Technologies) in a humidified 5% CO_2_ atmosphere at 37°C. Exponentially growing log phase cells were used in the present study. Normal human fibroblast cells, AG1521, were grown in α-MEM supplemented with 15% FBS and anti-anti. Only cells with passages lower than 12 were used. Contact inhibited G0/G1-phase cells were used for the experiments.

### Drug treatment

**Figure 1:**
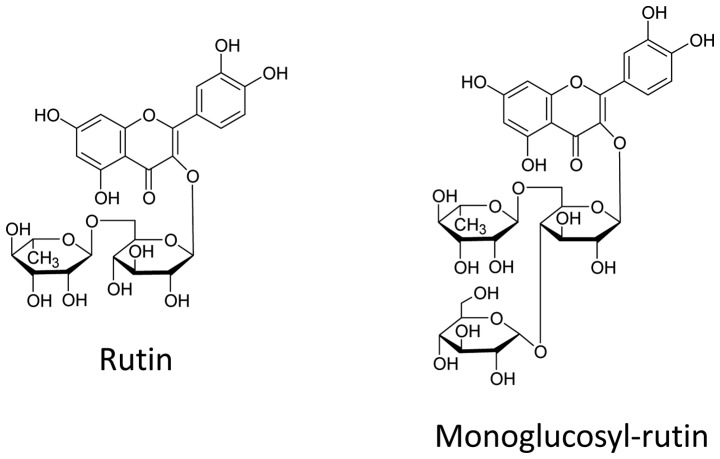
Chemical structure of rutin and monoglucosyl-rutin.

Monoglucosyl-rutin, an enzymatically modified form of rutin (Fig. 1), was supplied by Toyo Sugar Refining Co., Ltd. (Tokyo, Japan). Monoglucosyl-rutin was dissolved in phosphate-buffered saline (PBS) and filtered for sterilization. Monoglucosyl-rutin was freshly prepared for each experiment and was added to the cell culture 1 h prior to the beginning of the experiment.

### Irradiation

Irradiation was performed using a J.L. Shepherd Model Mark I-68 6000Ci ^137^Cs irradiator (J.L. Shepherd and Associates, San Fernando, CA, USA) at room temperature and at a dose rate of 250 cGy/min.

### Growth inhibition assay

To measure the cell doubling time, CHO cells were seeded at a density of 50,000 cells/P-60 cell culture dish with various concentrations of monoglucosyl-rutin, and the number of cells was counted twice a day (>8 h intervals) for 4 days. Cell doubling time for each condition was calculated using GraphPad Prism 6 software (GraphPad Software, San Diego, CA, USA). Three independent experiments were conducted.

### Cell survival assay

Cell survival was measured using a standard clonogenic assay. CHO cells were seeded at a density designed to yield ~100 viable colony-forming cells/P-60 cell culture dish subsequent to treatment with a combination of monoglucosyl-rutin and ionizing radiation. The colonies were scored 7–8 days after treatment. The dishes were then treated with 100% ethanol to fix colonies and stained with 0.1% crystal violet solution. The colonies containing more than 50 cells were recorded as reproductively viable surviving cells. Cell survival curves were drawn using GraphPad Prism software with linear quadratic regression. Three independent experiments were conducted.

### Immunostaining for γH2AX foci formation

The fixation and staining methods used for immunocytochemistry were performed as previously described (10). For immunostaining, AG1521 fibroblast cells were cultured on chamber slides and synchronized to the G0/G1 phases by contact inhibition. Following 30 min of treatment with monoglucosyl-rutin and radiation exposure at 37°C, the cells were fixed in 4% paraformaldehyde for 15 min, washed in PBS and then treated with 0.5% Triton X-100 for a further 10 min. Non-specific binding sites were inhibited using PBS with 10% goat serum at 4°C overnight. The chamber slides were then incubated with mouse monoclonal anti-γ-H2AX antibody (Millipore, Billerica, MA, USA) for 1 h, washed three times in PBS and incubated with Alexa 488 conjugated goat anti-mouse secondary antibody (Invitrogen Life Technologies) for 1 h at 37°C. The cells were washed four times in PBS and the nuclei were stained with Antifade Gold with DAPI solution (Invitrogen Life Technologies). Images of the cells were captured using a Z-stage motorized Zeiss Axioskop with Metamorph system (Carl Zeiss AG, Jena, Germany). The z-stacked images were stored and 50 cells from these were later scored in three independent experiments.

### Sister chromatid exchange (SCE) assay

CHO cells were synchronized into G1-phase by mitotic shake-off (11) and incubated for 2 h. Then, following treatment with monoglucosyl-rutin and radiation exposure, the cells were incubated with 10 μM BrdU for two rounds of cell replication. Colcemid was added and the cells were harvested and treated with 75 mM KCl, fixed with methanol:acetic acid (3:1) solution and placed onto slides. The slides were stained with Hoechst 33258 for 30 min, immersed in PBS and then exposed to a black light source at 55°C for 30 min. The slides were subsequently treated with 2X saline sodium citrate solution at 70°C for 30 min. Finally, the slides were stained with filtered 10% Giemsa solution in Gurr solution for 5 min. Metaphase images were obtained using an Olympus BX51 microscope equipped with a Q-imaging Aqua Cooled CCD camera with Q-capture Pro software (Olympus, Tokyo, Japan). The SCE frequency was scored for each chromosome. At least 50 metaphase cells were scored for each three independent experiments.

### Statistical analysis

Statistical comparison of the mean values was performed using a t-test with GraphPad Prism 6. P<0.05 was considered to indicate a statistically significant difference. Error bars indicate the standard error of the mean.

## Results

### Cellular toxicity of monoglucosyl-rutin

**Figure 2:**
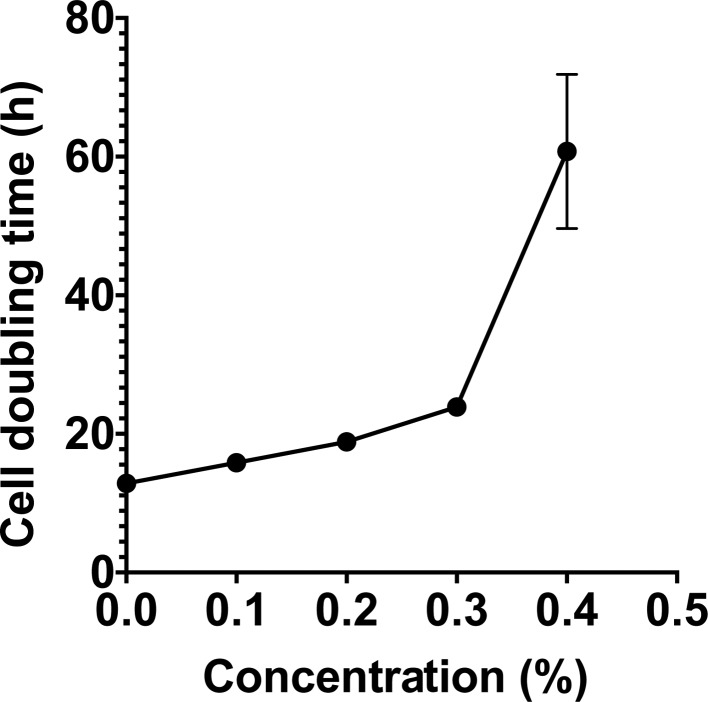
Cell doubling time of CHO10B2 cells exposed to various concentrations of monoglucosyl-rutin. Error bars indicate the standard error of the mean.

**Figure 3:**
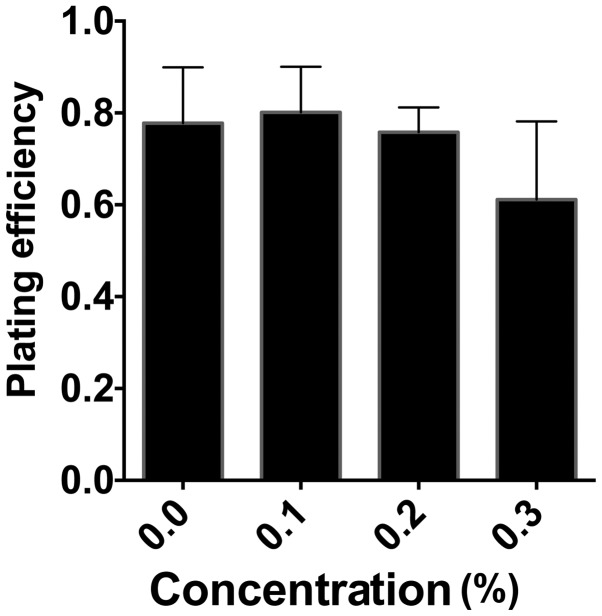
Cell plating efficiency of CHO10B2 cells exposed to various concentrations of monoglucosyl-rution. Error bars indicate the standard error of the mean.

To assess the cellular toxicity of monoglucosyl-rutin, the cell doubling time and plating efficiency of CHO cells were investigated. To investigate cell doubling time, CHO cells were treated with varying concentrations of monoglucosyl-rutin during growth. The total cell numbers were counted every 12 h to determine the cell doubling time for each treatment condition. As shown in Fig. 2, unexposed CHO cells exhibited an average doubling time of 12.8 h. Monoglucosyl-rutin significantly increased cell doubling time starting at concentrations as low as 0.1%. The IC_50_ for cells treated with monoglucosyl-rutin was 0.3% when the doubling time increased to 23.9 h. In addition to cell doubling time, the effect of monoglucosyl-rutin on cell plating efficiency was also investigated. CHO cells were treated with varying concentrations of monoglucosyl-rutin for 1 h and 100 cells were plated. Following seven days, the viable colonies were analyzed. As shown in Fig. 3, the plating efficiency of the treated cells was not significantly affected until 0.3% monoglucosyl-rutin was applied and the plating efficiency significantly decreased to 61%, compared with 77% in the control cells.

### Radioprotection in cell survival

**Figure 4:**
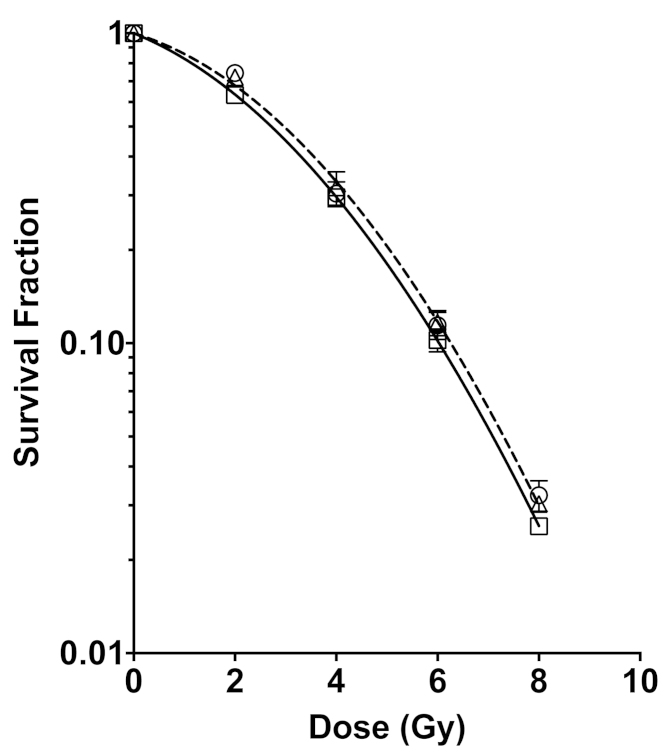
Cell survival of CHO10B2 cells at various doses of γ radiation. Open square indicates the control cells, open circle indicates cells treated with 0.1% monoglucosyl-rutin and open triangle indicates cells treated with 0.3% monoglucosyl-rutin. Error bars indicate standard error of the mean.

To assess the role of monoglucosyl-rutin as a potential radioprotector, cell survival was investigated using a colony formation assay. CHO cells were treated with 0.1 or 0.3% monoglucosyl-rutin for 1 h and then exposed to various doses of radiation. The irradiated cells were plated and the number of surviving cells was determined. As shown in Fig. 4, the cells treated with 0.1% monoglucosyl-rutin exhibited a statistically increased cell viability at 2 Gy when compared with the control cells. The cells treated with 0.3% monoglucosyl-rutin demonstrated a significant increase in survival at doses greater than 2 Gy.

### Radioprotection in DNA damage

**Figure 5:**
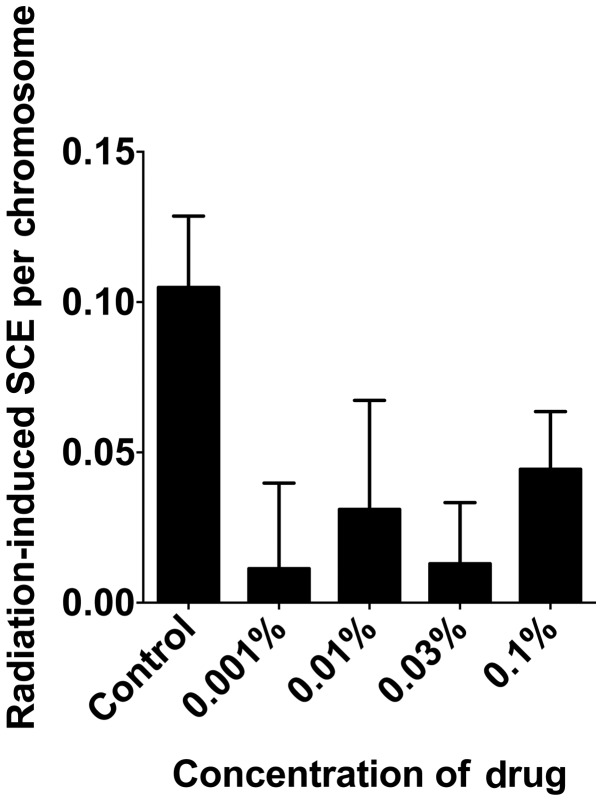
Number of radiation-induced SCEs per chromosome in CHO10B2 cells when exposed to 2 Gy of γ radiation. The error bars indicate the standard error of the mean. SCE, sister chromatid exchange.

**Figure 6:**
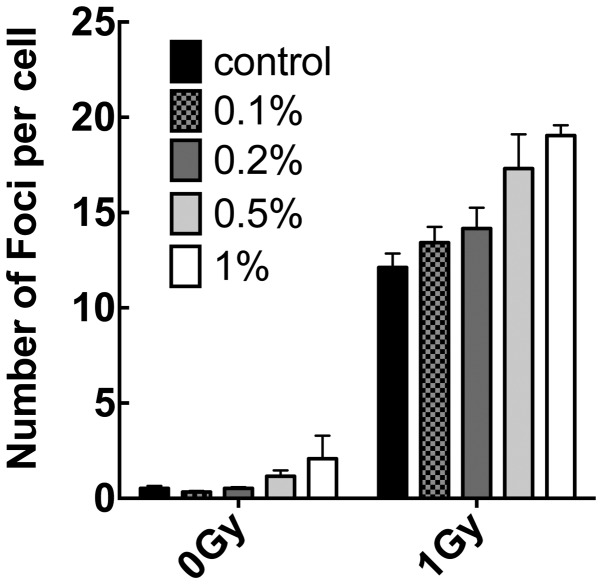
Average number of γH2AX foci/cell in normal human fibroblasts (AG1521) treated with various concentrations of monoglucosyl-rutin and exposed to 0 or 1 Gy of γ radiation. Treatment dose is indicated in the figure legend. The error bars indicate the standard error of the mean.

In order to determine the effect of monoglucosyl-rutin on the prevention of radiation-induced DNA damage, the number of induced SCEs and induced γH2AX foci were analyzed. To determine the effect of monoglucosyl-rutin on the prevention of DNA base damage, the number of 2 Gy γ-ray-induced SCEs were analyzed per chromosome. As shown in Fig. 5, monoglucosyl-rutin significantly decreased the number of SCEs per chromosome at all concentrations compared with non-treated cells. Additionally, the effect of monoglucosyl-rutin on double-strand breaks was also investigated by comparing the number of induced-γH2AX foci in treated and untreated cells. As shown in Fig. 6, at concentrations of 0.5 and 1%, monoglucosyl-rutin increased the number of γH2AX foci in unexposed and exposed fibroblasts. At lower concentrations used to determine cell survival and SCE, the monoglucosyl-rutin-treated cells exhibited statistically similar numbers of γH2AX foci with and without radiation.

## Discussion

The present study demonstrated that monoglucosyl-rutin exhibits a wide range of effects in mammalian cells. Despite the ability of monoglucosyl-rutin to protect cells from radiation at low concentrations, it was observed that at elevated doses cells experienced an increase in baseline DNA damage. These results are in accordance with previous studies demonstrating that natural flavonoids have beneficial and negative effects on cells (12). As demonstrated in a previous study, several flavonoids have been used to protect against radiation due to their ability to capture radiation-induced radicals *in vitro* and *in vivo* (7). However, it was also reported that certain flavonoids function as DNA-dependent protein kinase inhibitors (12).

Through colony formation assays it was demonstrated that at a concentration of 0.3%, monoglucosyl-rutin increases cell survival in cells exposed to 2 Gy. Concentrations of 0.1% monoglucosyl-rutin, however, only increased cell survival in cells exposed to 2 Gy but not 8 Gy. When the cells were analyzed for SCE and γH2AX foci, it was observed that all doses of monoglucosyl-rutin decreased the number of radiation-induced SCE events, however, had no effect on the number of γH2AX foci formed. These results suggest that monoglucosyl-rutin prevents DNA base damage caused by ionizing radiation. This indicates that monoglucosyl-rutin may inhibit long-lived radicals, however, not short-lived radicals which cause the most harm to cells, thus only a slight increase in cell survival was observed.

The limited effect of monoglucosyl-rutin on cell survival may also be attributed to the size of the chemical, which has a molecular weight (MW) of >700 Da. Compounds with a MW of >500 Da tend to not be absorbed through the cell membrane (13,14). This may potentially explain why monoglucosyl-rutin was found to be effective at inhibiting double-strand breaks *in vitro* in a previous study (15), however, demonstrated no effect *in vivo* in the present study.

Finally, it was noted in the present study that monoglucosyl-rutin had an effect on the plating efficiency and doubling time of the treated cells. Cell doubling time and plating efficiency were significantly affected at concentrations of monoglucosyl-rutin ≥0.3%. This is in accordance with the data which demonstrated that there was a statistical increase in γH2AX foci in cells treated with 0.5% monoglucosyl-rutin. Despite monoglucosyl-rutin being able to inhibit the formation of radiation-induced SCEs, the drug itself causes DNA double-strand breaks. These results demonstrated that there is a narrow window between doses that are potentially beneficial and ones that cause additional DNA damage to the cells.

In conclusion, the present study demonstrated that monoglucosyl-rutin is able to decrease the number of radiation-induced SCEs and increase cell survival at concentrations that do not significantly increase cellular toxicity. This increase in cell survival is possibly due to the ability of monoglucosyl-rutin to inhibit the formation of long-lived radicals, thus protecting against DNA base damage. At higher concentrations, however, it was revealed that treatment with monoglucosyl-rutin results in double-strand breaks, leading to increased cell doubling time and a decrease in overall cell survival, potentially limiting the use of monoglucosyl-rutin as a radioprotector. The present study demonstrated that *in vivo* exposure to monoglucosyl-rutin does not produce the same results as *in vitro* experiments, and this is most likely due to the inability of monoglucosyl-rutin to enter the cell owing to its large MW.
